# Understanding influences on physical activity participation by older adults: A qualitative study of community-dwelling older adults from the Hertfordshire Cohort Study, UK

**DOI:** 10.1371/journal.pone.0263050

**Published:** 2022-01-25

**Authors:** Jean Zhang, Ilse Bloom, Elaine M. Dennison, Kate A. Ward, Sian M. Robinson, Mary Barker, Cyrus Cooper, Wendy Lawrence

**Affiliations:** 1 MRC Lifecourse Epidemiology Centre, University of Southampton, Southampton, United Kingdom; 2 NIHR Southampton Biomedical Research Centre, University of Southampton and University Hospital Southampton NHS Foundation Trust, Southampton, United Kingdom; 3 Victoria University of Wellington, Wellington, New Zealand; 4 AGE Research Group, Translational and Clinical Research Institute, Newcastle University, Newcastle upon Tyne, United Kingdom; 5 NIHR Newcastle Biomedical Research Centre, Newcastle University and Newcastle upon Tyne Hospitals NHS Foundation Trust, Newcastle upon Tyne, United Kingdom; 6 NIHR Musculoskeletal Biomedical Research Unit, University of Oxford, Oxford, United Kingdom; The Education University of Hong Kong, HONG KONG

## Abstract

**Background:**

The health benefits of physical activity (PA) participation in later life are widely recognised. Understanding factors that can influence the participation of community-dwelling older adults in PA is crucial in an ageing society. This will be paramount in aiding the design of future interventions to effectively promote PA in this population. The main aim of this qualitative study was to explore influences on PA among community-dwelling older people, and the secondary aim was to explore gender differences.

**Methods:**

Qualitative data were collected in 2014 by conducting focus group discussions using a semi-structured discussion guide with older people resident in Hertfordshire, UK. Discussions were audio-recorded, transcribed verbatim and transcripts analysed thematically.

**Results:**

Ninety-two participants were recruited to the study (47% women; 74–83 years) and a total of 11 focus groups were conducted. Findings indicated six themes that appeared to affect older adults’ participation in PA: past life experiences; significant life events; getting older; PA environment; psychological/personal factors; and social capital. Overall, the findings emphasised the role of modifiable factors, namely psychological factors (such as self-efficacy, motivation, outcome expectancy) and social factors (such as social support and social engagement). These factors exerted their own influence on physical activity participation, but also appeared to mediate the effect of other largely non-modifiable background and ageing-related factors on participants’ engagement with PA in later life.

**Conclusion:**

In view of these findings, intervention designers could usefully work with behavioural scientists for insight as to how to enhance psychological and social factors in older adults. Our data suggest that interventions that aim to build self-efficacy, motivation and social networks have the potential to indirectly promote PA participation in older adults. This would be best achieved by developing physical activity interventions through working with participants in an empowering and engaging way.

## Introduction

Regular physical activity (PA) in older adults, those aged ≥65 years old, is robustly promoted by the World Health Organisation (WHO) [1]. Plethora of research have shown physical activity leads to risk reduction in a wide variety of co-morbidities such as type 2 diabetes mellitus [2], breast [3, 4] and colon cancer [3], and hypertension [5]. The range of PA advocated includes muscle strengthening, improving balance and 150 minutes of moderate intensity aerobic activity throughout the week. The updated UK Chief Medical Officer’s Physical Activity Guidelines published in 2019 echoes very similar messages to those of WHO [6]. However, given the emergence of new data indicating that a small amount of PA can show beneficial health effects [7, 8], the 2019 UK guideline has shifted away from recommending a minimum amount of time to exercise. In the section dedicated to people ≥65 years old, it emphasises “some physical activity is better than none: even light activity brings some health benefits compared to being sedentary” [6]. This shift in message may have a significant impact on how older adults view PA.

The level of PA participation in older adults is low; in 2016 the Health Survey for England reported that 67% of adults aged 19–64 years met the aerobic activity guidelines (71% of men; 63% of women) whereas only 44% of adults aged ≥65 years (48% of men; 41% of women) met the target [9]. Therefore, it is paramount to understand older adults’ perspectives on participation in PA which then can aid us to develop interventions to promote PA, thus translating its benefits into practice. Previous qualitative research into older adults’ perceptions of PA have tended to focus on structured PA, such as resistance training, balance and strength, and falls prevention programmes [10, 11] and has highlighted a number of influencing factors that can be largely categorised as: physical, social, environmental and psychological [10, 11]. Common facilitators include both physical and mental health benefits deriving from being physically active, and encouragement from peers, family and support networks [10, 11]. Frequently cited barriers to exercise include physical limitations due to pain or existing medical conditions [12, 13], and lack of motivation [13, 14]. A number of psychological factors have been strongly linked with higher levels of general PA and exercises in the older adult population, including high self-efficacy, positive thinking, and motivational internal thoughts [15–17]. In their review, Cavill and Foster focused on qualitative studies and previous reviews that investigated the barriers and facilitators to participation in strength and balance activities, having found specific motivators including reducing the risk or fear of falling, and preventing deteriorating and disability, and specific barriers including the perceived risk of a heart attack/stroke or death and fear of looking too muscular [10].

A UK qualitative study looked into older adults’ experiences of successful PA interventions and what would influence exercise adherence at a population level and found PA being “enjoyable, sociable, affordable, accessible, flexible and seasonal were more important than the health benefits” [18]. Other qualitative studies on PA in community living older adults have focused on various influences such as the impact of neighbourhood deprivation in New Zealand including environmental influences, such as the local community and levels of traffic [19], or cultural influences in older Mexican women living in the US, for example in terms of gender expectations [20]. There have been some studies on older adults’ perceptions of general PA, which can incorporate a wide spectrum of activities such as household chores, non-structured PA including walking, and hobbies [21–23]. In a recent systematic review of qualitative studies on older adults and PA, the principle finding suggests the biggest drive for PA is how it contributes to a purposeful and fulfilling life [24], rather than the health benefits. We had unique access to a group of UK community-dwelling older adults, i.e. older adults who were living in their own homes. The objective of this focus group study was to increase our understanding of factors that can affect older adults’ general participation in all types of PA and to identify potential opportunities for effective intervention to promote PA in this age group; a secondary aim was to explore any gender differences in factors affecting PA participation.

## Methods

### Participants

Participants for this study were selected from an established cohort, the Hertfordshire Cohort Study, comprising individuals born in Hertfordshire between 1931–1939 [25]. This cohort has previously been shown to be broadly representative of the wider population of older adults in England [26]. In 1998–2003 at baseline, 3,225 men and women had agreed to be interviewed at home. In 2011, 592 of these participants were approached for follow-up, of whom 443 (75%) were re-assessed. Detail on the background characteristics that were assessed at baseline and in 2011 has been published elsewhere [25]. Of these 443 participants, 408 (still alive and taking part in the study) were approached and invited to attend a focus group for the present study. Of the 408 invited, 92 (23%) participants agreed to take part in the study. The remainder (n = 316) did not take part due to various reasons, including unavailability in the study time frame, non-response to invitation letter or unwillingness. Full details of participant recruitment and the study procedure has been described elsewhere [27].

### Design

In brief, an invitation letter containing a participant information sheet and a reply slip were posted out to the participants. Telephone calls were then made to willing participants to arrange a convenient time to attend a focus group. The focus groups took place in a community venue in Hertford, UK between March and September 2014. Participants were reimbursed for their travel and refreshments were provided; no other incentive was offered. Each focus group was facilitated by one of the authors and was supported by another author acting as an observer, who made notes during the focus group discussions and debriefed afterwards with the facilitator (IB and WL). Both authors (female) had experience in qualitative methods. All groups were held separately for men and women in order to be able to explore gender differences, with the exception of one final group that combined men and women in order to fit with their availability. Ethical approval for this study was obtained from the NRES Committee East of England, Hatfield (REC reference: 10/H0311/59). The Hertfordshire Cohort Study had ethical approval from the Bedfordshire & Hertfordshire Local Research Ethics Committee and the West Hertfordshire Local Research Ethics Committee. All participants gave written informed consent before discussions began.

The focus group discussions followed a semi-structured guide to facilitate discussion of the study topics (see Supplementary material for a copy of the guide–S1 File). Semi-structured discussions maintain the focus of the discussion but allow for freedom and flexibility for participants to express their views [28]. Discussions were audio-recorded and transcribed verbatim. Topics covered included diet and PA; findings of the diet-related data have been published previously [27], and this paper now reports the PA data.

### Data analysis

PA data were analysed thematically [29] and independently of the analysis of the dietary data from the same participants [27]. The study adopted a critical realist position, assuming a realist ontology and a subjectivist epistemology, and thereby acknowledging that observations of reality and generation of knowledge are influenced by the theories and values that a researcher adopts [29, 30]. The transcripts were read, initial codes were identified to classify the data, and these were further organised into themes following the steps described by Braun and Clarke [29]. Initial codes were generated by double-coding of two transcripts; to give an example of how text was coded, a section of text talking about going dancing with friends would be coded under ‘social capital/social engagement’. All transcripts were double-coded by the two authors. Researchers compared their coding, combined their codes and organized them into themes to create an initial coding framework. This was used to double-code all transcripts, while being refined to show the emerging themes and categories from the transcripts. The process used was inductive coding whereby data were coded into themes and categories without the constraints of a pre-devised structure [29]. Each theme was analysed by two authors who met regularly to agree upon any divergences in coding and the categorisation of codes under relevant themes, to agree upon the coding framework, to discuss the analysis process, and how the themes might link together to provide insight into participants’ perspectives on PA and influences on their activity participation and habits. NVivo 12 qualitative data analysis software was used to assist with coding and data analysis.

## Results

### Focus group characteristics

Eleven focus group (FG) discussions were conducted with 92 participants, 47% were women, their mean age was 78 years, all were white British and living in their own homes. **Table 1** shows the descriptive characteristics of the study participants, as assessed in 2011. Discussions lasted between 75–99 minutes (mean = 90 minutes).

**Table 1:** Descriptive characteristics of focus group participants (n = 92) (data collected in 2011).

	N	Mean	SD
Gait speed (m/s)^1^	91	0.78	0.16
Grip strength (kg)^2^	91	30.1	10.1
Height (cm)	91	167.2	8.5
	**N**	**Median**	**IQR**
Weight (kg)	92	78.3	71.5–86.9
BMI (kg/m^2^)	91	27.7	25.4–30.8
Alcohol consumption (units per week)	92	3.5	0.8–10.7
Activity time in last 2 weeks (min/day)^3^	85	199	147–283
	**Total N**	**N**	**%**
Age left education (age at leaving full-time education)	92		
< = 14		14	15.2
> = 15		78	84.8
Social class	89		
I-IIINM (professional, managerial/technical and skilled non-manual occupations)		43	48.3
IIIM-V (skilled manual, partly skilled and unskilled occupations)		46	51.7
Smoker status	92		
Never		45	48.9
Ex		44	47.8
Current		3	3.3
Number of comorbidities^4^	92		
0		18	19.6
1		37	40.2
2		25	27.2
3		7	7.6
4 or more		5	5.4

^1^ Gait speed was ascertained using the mean time from two 8ft gait speed tests.

^2^ Grip strength was assessed three times for each hand using a Jamar dynamometer; the highest measurement was used for analysis.

^3^ Level of physical activity was assessed using the Longitudinal Ageing Study Amsterdam Physical Activity Questionnaire (LAPAQ).

^4^ Number of comorbidities out of: bronchitis, diabetes, ischaemic heart disease, hypertension, stroke.

### Thematic analysis

Six overarching themes were identified through the process of thematic analysis: (1) past life experiences, (2) significant life events, (3) getting older, (4) physical activity environment, (5) psychological/personal factors and (6) social capital.

These themes are described below, with quotations offered as illustrative examples in **Table 2**. Quotations are labelled with the number of the focus group and M or W to indicate whether the speaker was a man or woman. We present the findings as they address the primary objective of the study which was to explore what influences physical activity (PA) participation among community-dwelling older people, and where applicable note differences between men and women.

**Table 2:** Quotations illustrating each of the six themes.

Themes	Quotations
**1. Past life experiences**	
Quotation 1	*‘I’ve been used to all my life a fair amount of physical activity*, *an active life and I suppose it comes from a fairly hard working life*. *Since I retired many years ago I tried to expand that a little’ (FG11MW)*
Quotation 2	*‘Well I’ve got three dogs and I walk for an hour every day and I swim*. *I was a swimming teacher for 28 years*, *so I am pretty active…’ (FG4W)*
Quotation 3	*‘W1*: *… I used to play badminton 3 times a week and table tennis*, *as well as the swimming*, *I don’t do as much now … it’s made me too tired…* *W2*: *Haven’t got the energy have we*, *like we used to have’ (FG6W)*
**2. Significant life events**	
Quotation 4	*‘M1*: *Once you’ve retired you gotta get out and do*, *you gotta go* *M2*: *You gotta keep going* *M3*: *I get up at the same time as when I went to work’ (FG10M)*
Quotation 5	*‘The moment I retired I had a terrific change in life because my job was 24/7 seven days a week and I left it all behind … I’m president of the sports club which runs three football teams*, *two cricket teams and I get involved in a lot of the village life …’ (FG10M)*
Quotation 6	*‘… I worked all my life seven days a week most of it*, *and then to suddenly stop work and you find … all the days are the same and you know*, *you get very lazy actually because ‘oh I won’t do it this morning I’ll do it this afternoon’…’ (FG5M)*
Quotation 7	*‘I recently lost two friends*, *two brothers and a son-in-law*, *I think that has caused the depression*.*’ (FG7W)*
Quotation 8	*‘W1*: *… I then joined a women’s club*, *things like that*, *to get myself out in the first place … being in the company of other people that are in the same position … must give you a bit of confidence really*. *W2*: *You do find that whereas we were … quite a bit*, *out and joining other couples*, *you did find that you weren’t invited so much (noises of agreement)’ (FG7W)*
**3. Getting older**	
Quotation 9	*‘…I try to walk but I can’t walk you know*, *since I had the heart operation’ (FG6W)*
Quotation 10	*‘… as we’re getting older most of us have either got a bad knee or a bad hip*. *In my case I used to do a lot more walking than I do now*, *but I just can’t do*, *it’s too painful…" (FG5M)*
Quotation 11	*‘It’s through back and knees … but you know*, *I’m lucky I can still keep walking and … I think you learn to live with pain … I think there are very few old people who don’t have pain*, *and I think it’s the degree of it*, *but you just have to ignore it a bit’ (FG9W)*
Quotation 12	*‘Yes*, *I think I still do the same things but a little slower’ (FG11MW)*
Quotation 13	*‘I had a bit of blood pressure and I went to the doctor’s and they said ‘keep up the bowling*, *it’s good exercise for you’… so I keep doing it’ (FG3M)*
Quotation 14	*‘The doctor said … ‘you’ll have to pack up working’ I said ‘I can’t’… and I thought well I’m gonna prove you wrong … I had to talk the surgeon into doing two of mine at the same time*, *never done it he said before … Went back after six weeks … without crutches or walking sticks or anything and everybody was still on their crutches and they had one done*, *so he said ‘how did you do that’ I said ‘determination’ (FG10M)*
**4. Physical activity environment**	
Quotation 15	*‘A tremendous variety of activities and one of them is the walks we go on once a month*, *but they have longer walks*, *they have eight mile walks*, *ten mile walks*, *fast walks*, *slow walks … ‘ (FG7W)*
Quotation 16	*‘I mean an awful lot of people of our age group have never heard … ‘U3A what is that’ you know and I would’ve thought they would’ve at least heard of it …’ (FG9W)*
Quotation 17	*‘But these swimming pools … they don’t make the time for the older people to have their time and you don’t really want to go with youngsters splashing*, *you just want to go in and have swim and that’s it*, *but you feel sometimes that they’re not catering for our generation…’ (FG6W)*
Quotation 18	*’… I know one or two people who actually*, *you know they get up in the morning and on goes the television and that’s their day*, *because they can’t really afford to do anything else’ (FG10M)*
**5. Psychological/ personal factors**	
Quotation 19	*‘Q So what motivates you to keep up that level of physical activity*? *[moderator’s question is followed by discussion by participants] Q How do other people feel about that*, *about being busy*? *[answers are given by multiple participants]*
	*A [I’m] up ladders and sticking windows up …*
	*A When you stop doing something then you never take it up again*, *so you got to keep doing what you’ve been doing all your life’ (FG8M)*
Quotation 20	*‘[In the context of discussion prompted by moderator about what local services/support might be necessary to support people to undertake PA]*
	*A You’ve got to stay healthy and fit…*
	*A I think our opportunities are quite good actually if you’ve got the motivation to go out and find them…’ (FG1W)*
Quotation 21	*‘… I got this belief that if I’m doing the marathons … I’ll still be able to do all the things that I do … the minute I slow down*, *and walk ‘round Tescos with a trolley*, *then I won’t be able to do the things*, *that’s why I keep doing it*. *I sort of have got the belief that if I did it today*, *I can do it tomorrow and so I try and do it every day’ (FG8M)*
Quotation 22	*‘For me*, *a healthy lifestyle is being able to take part in things*, *still able to enjoy new activities and just generally not feel old actually (laughs) … people say ‘Oh*, *you’re doing very well for your age’ and I say ‘Well what am I supposed to be able to do at my age*?*’ (laughs)’ (FG4W)*
Quotation 23	*‘If you didn’t do any exercise it would seize up*, *wouldn’t it*, *for good perhaps’ (FG9W)*
Quotation 24	*‘… I still get on the bike and also the treadmill but not as long as I should do’ (FG5M)*
Quotation 25	*’…I’m not at all keen on this group thing*, *I’d rather do things on my own with my wife and family…’ (FG5M)*
Quotation 26	*‘I go line dancing twice a week*, *so that’s my exercise*, *I would never go to a gym (laughs)’ (FG9W)*
Quotation 27	*’… You feel as if you’re not sort of educated enough to join the group*, *because … you find lots of very professional ex-headmistresses and that*, *you know what I mean*, *I feel inferior’ (FG3M)*
**6. Social capital**	
Quotation 28	*’*.* *.* *.*So*, *you see other people’s lifestyles and you think to yourself ‘well they don’t look quite as fit*, *so I need to keep going” (FG3M)*
Quotation 29	*’I’ve got one or two friends with*.* *.* *. *Alzheimer’s disease and Parkinson’s and it worries me*.* *.* *. *that could be me one day*.* *.* *. *and I think at the back of your mind that you’re trying to keep yourself in as good a shape as you can*.* *.* *.*’ (FG2M)*
Quotation 30	*‘…The dog insists on me taking it for a walk (noises of agreement) I’m not sure which one of us takes which (laughter)…’ (FG11MW)*
Quotation 31	*’…My wife who died eighteen months ago*, *she had to walk because she had a problem with her leg circulation*.* *.* *.*so that encouraged me to walk as well*, *so we did a lot of walking together’ (FG5M)*
Quotation 32	*‘My husband comes with me as well*, *he’s 90*, *he does exercise*, *he’s better than me (laughs)’ (FG7W)*
Quotation 33	*’I’ve got two neighbours either side of me with young children … and they’re great they’re really’ (FG1W)*
Quotation 34	*’I think the friends are very important*.* *.* *.*it’s sometimes more important than family (noises of agreement) because you can’t alter their lives*, *they’ve got their own lives to live’ (FG7W)*
Quotation 35	*‘I*, *for instance*, *have a friend… she and I both lost our husbands in the same year… I don’t think I would’ve done half the things that I did if she wasn’t there for company (noises of agreement) and I think if you can get two or three people who are alone together to go along things*, *rather than leaving them on their own’ (FG7W)*

#### 1. Past life experiences

Many participants spoke of PA habits or routines from previous life stages, e.g. early life or working life, continuing into older age (quotations 1 and 2). Others had stopped being active in the past for a variety of reasons such as busy lifestyles (e.g. work or raising a family), a new medical condition or event that impeded PA participation, and had not resumed their activity later in life, or because of they had aged (quotation 3).

#### 2. Significant life events

This theme includes major events that are common in later life, which may present particular challenges to older people’s daily routines and habits.

*2*.*1*. *Retirement*. In this cohort of older adults, men were more likely to mention retirement, than the women. This perhaps reflects the generation where the men tend to be the main earner for the family than the women. Therefore, it was noted the men were more likely to mention having more free time or freedom since retirement to participate in pursuits, take up new hobbies, join new organisations, or volunteer. They spoke of the importance of keeping going after retirement and maintaining a routine (quotation 4). Although some enjoyed a busy schedule after having made a complete break from their working life (quotation 5), others had difficulty adapting to the lack of structure post-retirement (quotation 6).

*2*.*2*. *Bereavement*. For both men and women, adjusting to losing a partner included taking up activities that they previously had not done. There were some gender differences in this regard. Men spoke of doing new activities such as housework. For women, losing their husband led to more walking if they did not drive, or less if they used to walk together. Loss of a partner, pet, friend or family member also led to feelings of isolation and loneliness, with some suffering a loss of confidence, or depression (quotation 7); participants spoke of these aspects in the context of discussion around their views on a ‘healthy lifestyle’ and their PA habits, so it appeared likely that they affected their PA, even if in an indirect manner. Women highlighted the importance of having a supportive network, for example someone going through similar experiences (quotation 8).

#### 3. Getting older

Debilitating health conditions, e.g. joint or heart problems, and the resultant medical events, e.g. hip or chest operations, impacted on activities, physical condition and loss of abilities such as driving or walking (quotation 9). Additionally, some medications caused side-effects like drowsiness. The decline in capability or ability to do certain activities, slowing down due to the natural ageing process and deconditioning of musculoskeletal health were important constraining influences on physical activity (quotation 10).

While some felt physically limited due to a heart condition, for instance, or a major medical event, others were determined to get back to their original capacity and not be prevented from doing the PA they wanted to do. In some cases, a medical condition or event had led them to do specific exercises or to adapt types of PA to suit their current condition (e.g. rehabilitation exercises). Pain and discomfort were barriers to doing PA, with some determined to ‘walk through it’ (quotation 11). Many spoke of maintaining activities despite being stiff and suffering general decline, of slowing down the pace but still continuing to complete the task (quotation 12). Interactions with medical professionals encouraged PA, including some wanting to prove medical professionals wrong (quotations 13 and 14).

#### 4. Physical activity environment

*4*.*1*. *Availability of local services and facilities*. The discussions revealed varying knowledge with regards to availability of local facilities and activities, for example, some took advantage of regular activities such as walks, some had never heard of U3A (University of the Third Age) and for others it was an important part of their lives (quotations 15 and 16). The U3A is an organisation of locally-run interest groups around the UK that provide opportunities for members, usually individuals who are no longer in full-time work, to engage with others to undertake a broad range of activities together. Some women spoke of a lack of publicising of local organisations and facilities, and women were more likely than men to speak of reduced opportunities suitable for older people, as illustrated in quotation 17, where some felt there was a lack of certain facilities suitable for older people.

Having access to a suitable environment at home or nearby was a motivator for PA, e.g. having access to a garden, parks, and allotments. Some localities had greater availability of facilities than others, and there was mention of a loss of green spaces.

*4*.*2*. *Accessibility*. Availability of transport and having a bus pass offered freedom and pleasurable experiences. While some were reliant on a bus pass, others rarely used it. Some mentioned a lack of local transport, and how not being able to drive was restrictive. Affordability of activities and transport was an issue for some and could mean reduced participation in activities (quotation 18).

#### 5. Psychological/personal factors

*5*.*1*. *Mental state*. For some participants, their mental well-being, low mood/depression and self-esteem appeared to be important aspects relating to the activities that they felt able to do. Participants also spoke widely of the enjoyment of activities, and the importance of keeping not only physically but mentally and socially active.

*5*.*2*. *Motivation and maintaining independence*. Discussions highlighted the importance of determination, discipline, positive thinking, motivation, independence, and self-efficacy (a belief in one’s ability to undertake an action). There was a strong desire in some not to be restricted, and to keep going in order to remain independent; there was an awareness of ageing, with a fear of stopping being active as it might then be harder to restart (quotations 19, 20 and 21).

*5*.*3*. *Beliefs and self-awareness*. Many were determined to take part in things, to ’not feel old’, and did not want to be perceived as incapable purely due to chronological age, and the importance of interacting with people of all ages as ’that keeps you young’ (quotation 22). Some demonstrated ‘outcome expectancy’ (anticipation of future outcomes as a result of current actions) regarding PA being beneficial for long-term health or longevity (quotation 23). Some mentioned a lack of motivation, perceived as laziness, as a barrier to PA, acknowledging that they ‘should’ do more (quotation 24).

Personal preferences affected engagement with certain types of PA, such as disliking group settings or organised activities, and some suggested alternatives that they would prefer (quotations 25 and 26). Some men said they felt unworthy in some way of joining group activities (quotation 27).

#### 6. Social capital

Many participants were involved in local or community groups and organisations (e.g. village committees, volunteering, U3A), although some of these were more mentally stimulating than physically challenging. Social aspects of PA and lifestyle more generally were important. Social comparisons were made with peers’ health and PA behaviour. It appeared that behaviours were sometimes motivated by fear resulting from others’ experiences or feeling fortunate to have relatively good health compared to others (quotations 28 and 29). Having a dog was an important motivator for some participants to do PA, particularly as it appeared to function as an incentive to go for walks on a regular basis (quotation 30); moreover, the social aspects of dog walking, including meeting other dog walkers and walking and talking together, appeared to be an important factor for engagement in this activity. Family, friends, and partners were important motivators (quotations 31 and 32) and as a source of social support. However, in some cases they could have a negative impact on PA due, for example, because their role as carers constrained their time. For both men and women, living alone was a potential barrier to social interaction and PA. Women were more likely than men to mention the importance of having helpful or supportive neighbours and community (quotation 33). For some this aligned with a desire to avoid being a burden on family which appeared to be a motivation to keep fit (quotation 34). The role of friends appeared to be particularly important for those who had lost their partner or lived alone, and there was suggestion that activities that are not just couple-oriented might be of importance (quotation 35).

## Discussion

This study has highlighted six themes that might affect community-living older adults’ participation in PA: past life experiences; significant life events; getting older; PA environment; psychological/personal factors; and social capital. The first three are largely non-modifiable as they tend to be events that have already happened or over which there is potentially limited or no control, however how an individual responds to these events could be modifiable. The opportunity to optimise the PA environment is likely to require a broader intervention approach and involvement of a multitude of stakeholders, such as local authorities, in order to make meaningful changes. Our analysis proposes the remaining two factors–psychological/personal and social capital partly determine how people respond to largely non-modifiable factors. For instance, those with higher levels of self-efficacy or determination, might be more likely to respond positively to the consequences of ageing and medical events or conditions, by maintaining or increasing their PA. There was a gender difference in response to the loss or serious illness of a partner. Women who had access to and utilised their social network appeared to be more likely to remain engaged in PA. Whereas for men, the loss or serious illness, of a partner led to the need to carry out more household chores, but not to a specific interest in engaging with social activities. Despite approaching the PA data inductively, the same themes and relationships between the themes appeared to hold for the PA data as they did in the published diet findings in this group [27]. Unhealthy lifestyle behaviours including poor diet and low PA levels often co-occur in older adults [31, 32], and we can speculate that the motivations might be similar for both types of behaviour.

The role of psychological and social factors has been identified in previous quantitative studies that have shown leisure time PA to be significantly linked with self-efficacy [15] and improved social, physical, emotional and cognitional function [33]. Specifically, thinking more positively and having motivational internal thoughts have been extensively shown to be associated with increases in older adults’ leisure time PA [15–17]. In a systematic review of 63 studies with adults aged ≥60 years [17], motivation and self-efficacy were the two psychological characteristics that were consistently associated with higher level of routine PA [17]. In our focus groups of community-dwelling older adults we observed variability in psychological and social factors that can affect the response of an older person to an event. This poses an interesting avenue for intervention targets, with health coaching using elements of motivation and self-efficacy to promote healthy behaviours. There is great heterogeneity in what constitutes health coaching [34]. It commonly consists of patient-centred approaches based on a behavioural change model that is delivered by a trained professional. The process often involves patient-determined goals, self-discovery and accountability. The effects of health coaching on level of PA participation in those aged ≥60 years have been found to be small but significant, compared to controls, and it is effective in both older adults with and without chronic conditions [35]. The use of “positive framing” has also been used in older adults in the context of walking; in one intervention study those exposed to “positive framing”, where participants were informed of beneficial effects of walking, were found to walk more steps, after controlling for baseline number of steps walked, than those receiving “negative framing”, where participants were informed of negative effects of not walking [36]. In a weight-loss trial, a motivation-focused programme that specifically targeted motivational factors showed comparable weight loss to the traditional skills-based weight loss programme [37].

The presence of physical limitations or health-related conditions has been found to be closely related with older adults’ perceptions of being able to be physically active [11, 24]. Interestingly in our study we found polarising views on this matter. Some felt that their health-related issues were a barrier to being active, whereas others refused to let medical conditions be an impediment to their participation in PA, therefore making particular efforts to overcome obstacles. Halvarsson and colleagues interviewed older women (≥65 years), with a diagnosis of osteoporosis and either self-perceived fear of falling or who had experienced a fall within the past 12 months, after participating in a balance training programme [38]. The aim of the programme was to improve self-efficacy, balance and physical function [39]. The programme improved participants’ self-perceived empowerment and self-efficacy, therefore resulting in the ability to approach activities of daily living with confidence and to employ risk-reducing strategies to avoid falls. However, the participants’ assessment of risk still remained influenced by their own perception of fragility [38]. Aligning with our study findings, this reflects how some people’s perception of their health is a barrier to be overcome, whereas for others it is insurmountable.

### Strengths and limitations

One of the limitations of this study is the age range of 74–83 years, which was pre-determined by the age range in the Hertfordshire Cohort Study. This does not incorporate the full age range that is generally included in the older adults’ category (i.e.: ≥65 years). Although data were collected in 2014, there is no reason to believe that findings would be different if we had conducted the research more recently. It should be noted that in the current context of the ongoing COVID-19 pandemic, increasing evidence indicates a decrease in PA levels of older adults due to related restrictions [40, 41]. Only white British adults participated in the focus groups, so we have no insight into any additional factors that could be relevant to other ethnic groups. However, a key strength of this study is the wide range of socio-economic backgrounds represented, therefore findings may be relevant to a large proportion of the UK older population.

We acknowledge that this study presents only one view, and other interpretations are possible. However, the analysis was conducted independently by two researchers through a rigorous process of double-coding, thereby reducing the probability of skewed views being presented. Throughout the study process, a multidisciplinary team approach was utilised, therefore minimising the probability of misrepresentation.

### Implication for future research and practice

A variety of modifiable psychosocial factors, including self-efficacy, motivation, outcome expectancy, and social support and engagement, appeared to influence older adults’ PA. Specifically psychological and social factors affected how older people overcame historical, environmental, medical and other ageing-related barriers, in order to incorporate PA in their lives. Therefore, this presents possible avenues for targeted interventions for older adults in encouraging PA.

Our findings regarding the importance of having a supportive network, including friends and neighbours, particularly for women who had lost their partner or lived alone, suggest that interventions to promote PA should take these aspects into account in their design, e.g. ‘buddy-up’ schemes to promote engagement with activities.

Furthermore, the physical environment was another modifiable element highlighted in our study; optimisation of the PA environment is important and without a facilitative infrastructure to propel meaningful progress, individual change can be challenging. Our study’s findings suggest that involving older adults should be a key aspect of any intervention to enhance the local PA environment, as older adults could offer invaluable insights into local need and could work together with local councils to improve transport links and green spaces, for example.

This highlights the value of adopting participant-led approaches when designing interventions, emphasising the need to include older adults through user-led approaches, and be aware that a “one size fits all” approach would not be appropriate. Those who are likely to use such programmes are best placed to be able to provide insights into factors that are important to their age group [42, 43]. Co-production of interventions promotes a sense of ownership. These findings also emphasise the importance of involving behavioural scientists from the beginning of intervention programming. They understand not only theories of behaviour change, but can identify ways to (i) enhance self-efficacy (e.g. by providing mastery experiences), (ii) utilise effective behaviour change techniques (such as social support, goal-setting, prompts and cues, role models), (iii) increase sense of control (e.g. by involving older adults in public patient involvement), and (iv) motivate and empower using established programmes. More recent physical activity interventions have begun to incorporate the perspectives of the intended users such as “The Move for Life Study” [44] and the “Osteoarthritis Physical Activity Care Pathway”[45].

Research is required to further explore how the COVID-19 pandemic has impacted community-living older people’s health, well-being and PA behaviour. However, we believe that the data from the present study could still provide useful insights into how an ageing population view physical activity and how best to intervene to increase participation.

## Conclusion

In an ageing population, healthy living is becoming a key concept and this has thrown up new challenges, thus to facilitate the adoption and maintenance of PA the inclusion of the intended users’ perspectives is crucial. This study sought the viewpoints of a group of free-living community older adults who had not been recruited for specific PA related activities or programs. Consequently, PA in its broadest sense was discussed psychosocial factors emerged as key influences, suggesting that interventions that target factors such as self-efficacy and social engagement, might hold potential for promoting engagement in general PA. These findings would aid the development of future interventions and as well as local authority planning for the needs of older adults living in the community.

## Supporting information

S1 File Focus group discussion guide. (DOCX)

S2 File COREQ checklist. (PDF)
