# Supplementary material for: Understanding influences on physical activity participation by older adults: A qualitative study of community-dwelling older adults from the Hertfordshire Cohort Study, UK
Source: PLoS One. 2022 Jan 25;17(1):e0263050. doi: 10.1371/journal.pone.0263050 (PMC8789143; doi:10.1371/journal.pone.0263050)
Supplement: S1 File — (DOCX) [file pone.0263050.s001.docx]

**Hertfordshire Qualitative Study – Focus Group Discussion Guide**

**Main research aim:** To gain a better understanding of the factors that influence food choice, diet quality and physical activity in older adults.

**Welcome, thank you and housekeeping:**

(tape-recording, fire exits/alarms, toilets, mobile phones, timings for the day)

**Introduction:** ‘This study is interested in the lifestyle choices of older adults, in order to consider how they might be supported to maintain or improve their overall health. The purpose of the discussion today is to learn more about your food choices and physical activity.’

**Introduction & opening question:**

‘Can you tell us who you are and say what a healthy lifestyle means to you?’

**Questions (diet):**

- What foods do you enjoy, eat often, rarely or never?
- What do you think influences your food choices? *(Refer to prompt list if necessary)*
- What prevents you from choosing other (healthier?) foods? *(Refer to prompt list if necessary)*
- How do you think your diet has changed over the last 10-15 years?
- What caused those changes to happen?

**Questions (physical activity):**

- What sort of physical activity do you do (in leisure time, structured or not, frequency…)?
- What motivates you to exercise? *(Refer to prompt list if necessary)*
- What do you think prevents you from doing more (any) physical activity? *(Refer to prompt list if necessary)*
- How do you think your exercise habits have changed over the last 10-15 years?
- What caused those changes to happen?

**Questions (general):**

- What do you think could help people maintain a healthy lifestyle as they get older?
- In order to help people achieve this, what services/initiatives do you think should be added/improved in the community?
- How would you feel about taking part in a lifestyle-focused intervention study?

**Ending questions:** We are almost finished with our discussion:

- What, if anything, have we not covered?
- What, if anything, would you like to add?

**Prompts – Possible influences on food choice/diet quality in older people:**

| Dietary knowledge | Past experiences | Habit | Cooking skills |
| --- | --- | --- | --- |
| Social setting | Social support | Eating alone | Loss of a partner / loneliness |
| Physical and mental health / wellbeing | Weight concerns | Appetite | Taste |
| Priority of food | Transport | Access | Money |
| Self-efﬁcacy |  |  |  |

**Prompts – Possible influences on physical activity in older people:**

| Knowledge | Disease Management | Advice from healthcare professional | Physical and mental health / wellbeing |
| --- | --- | --- | --- |
| Fear and Negative Experiences | Social Isolation | Social Activity | Environment |
| Time | Money | Lack of Interest | Self-Efficacy |
